# Distinct Role of Kruppel-like Factor 11 in the Regulation of Prostaglandin E_2_ Biosynthesis*

**DOI:** 10.1074/jbc.M109.077065

**Published:** 2020-10-23

**Authors:** Navtej S. Buttar, Cathrine J. DeMars, Gwen Lomberk, Sumera I. Ilyas, Juliana Bonilla-Velez, Shalini Achra, Shahrooz Rashtak, Kenneth K. Wang, Martin E. Fernandez-Zapico, Raul Urrutia

**Affiliations:** ‡ From the Division of Gastroenterology, Rochester, Minnesota 55902; § Schulze Center for Novel Therapeutics, Mayo Clinic, Rochester, Minnesota 55902

**Keywords:** Chromatin/Remodeling, Chromatin/Epigenetics, Eicosanoids/Prostaglandins, Lipid/Phospholipases, Transcription/Zinc Finger, Carcinogenesis, Esophagus, Inflammation

## Abstract

Kruppel-like factor (KLF) proteins are emerging as key regulators of lipid metabolism, diabetes, and the biosynthesis of immunological cytokines. However, their role in the synthesis of prostaglandins, widely known biochemical mediators that act in a myriad of cell biological processes remain poorly understood. Consequently, in this study a comprehensive investigation at the cellular, biochemical, and molecular levels reveal that KLF11 inhibits prostaglandin E_2_ synthesis via transcriptional silencing of the promoter of its biosynthetic enzyme, cytosolic phospholipase A2α. Mechanistically, KLF11 accomplishes this function by binding to the promoter via specific GC-rich sites and recruiting the Sin3-histone deacetylase chromatin remodeling complex. Further functional characterization reveals that this function of KLF11 can be reversed by epidermal growth factor receptor-AKT-mediated post-translational modification of threonine 56, a residue within its Sin3-binding domain. This is the first evidence supporting a relevant role for any KLF protein in doing both: transcriptionally inhibiting prostaglandin biosynthesis and its reversibility by an epidermal growth factor receptor-AKT signaling-mediated posttranslational mechanisms.

## Introduction

Prostaglandin E_2_ (PGE_2_)^2^ biosynthesis pathway is strongly implicated in wide-ranging physiological and pathological events, such as ovulation, implantation, and parturition during reproduction; ductus arteriosus closure in neonates; pain hypersensitivity, inflammation, febrile response, gastric mucosal protection, T cell differentiation and repair during acute or chronic injury; cell proliferation, neoplastic transformation, and invasion during carcinogenesis as well as alteration in kidney function, vascular tone, bone resorption, and neurological disorders like Alzheimer disease. Therefore expanding our understanding of regulation of PGE_2_ biosynthesis has unique biochemical and cellular relevance (1).

The regulation of the PGE_2_ synthesis pathway can be divided into three main steps, in which a key step involves the mobilization of arachidonic acid from membrane phospholipids by the action of phospholipase enzymes (2, 3, 4, 5). Among many phospholipases, the cytosolic phospholipase A2α (*cPLA2*α) has very high substrate specificity toward arachidonic acid, and therefore is the key regulator of intracellular arachidonic acid release (2, 3, 4, 5, 6). The mobilization of arachidonic acid is generally considered the rate-limiting step in the synthesis of PGE_2_ (4). Subsequent steps in the PGE_2_ synthesis pathway involve the alignment of COX-2 and microsomal PGE_2_ synthase, which convert intracellular arachidonic acid to a prostaglandin intermediate and finally PGE_2_ (7, 8, 9). The intracellular release of arachidonic acid, which occurs via *cPLA2*α, favors COX-2 and microsomal PGE_2_ synthase to synthesize PGE_2_ instead of other prostaglandins (8, 9, 10). Moreover, *cPLA2*α-mediated arachidonic acid release is also known to increase COX-2 promoter activity, as well as protein synthesis, thereby redirecting arachidonic acid toward PGE_2_ synthesis (11). Altogether, the overall process of PGE_2_ synthesis is highly complex and remains to be further investigated at the transcriptional level.

In the past decade, cyclooxygenase, particularly COX-2, has received significant attention for their importance in prostaglandin biosynthesis. Several elegant reports have provided evidence that COX-2 inhibition and consequent down-regulation of PGE_2_ synthesis have important therapeutic implications in many pathological conditions, such as inflammation and carcinogenesis (12, 13, 14, 15). However, in recent investigations, it has been recognized that long-term COX-2 inhibition is not safe due to increased cardiac and cerebrovascular side effects (16, 17, 18). The explanation for such complications in the setting of COX-2 inhibition is an ongoing production of arachidonic acid by phospholipase A_2_. This arachidonic acid is then utilized by competing homeostatic pathways, which results in the relative change of certain eicosanoid levels, to cause these side effects (19, 20, 21, 22). Similar to COX-2, the importance of *cPLA2*α has been confirmed *in vivo* via genetic experiments that show a reduction in levels of PGE_2_ and polyposis in APC min/+ with either mutation in or deletion of the *cPLA2*α locus (23, 24). In stark contrast to COX-2 antagonists, the inhibition of *cPLA2*α decreases the overall production of arachidonic acid, thereby preventing misdirection of the PGE_2_ substrate to competing homeostatic pathways that cause cerebrovascular toxicity. In fact, transient middle cerebral artery occlusion in *cPLA2*α^−/−^ mice results in smaller infarcts, and these mice develop less neurological deficits (5). Therefore, *cPLA2*α is a remarkable alternative candidate node instead of COX-2 inhibition to target the PGE_2_ synthesis pathway and thereby expand our basic understanding of prostaglandin biosynthesis that is central to cellular homeostasis and the pathobiology of many diseases.

KLF proteins have elicited significant attention due to their emerging key regulatory roles in several cellular functions (25, 26, 27, 28, 29, 30, 31, 32). Our laboratory has been studying the role of KLF proteins, in particular KLF11 and KLF13, in both lipid and glucose metabolism (33, 34, 35). In the current study, we performed extensive characterization of the role of KLF11 as a key mediator of prostaglandin biosynthesis and report that the expression of KLF11 reduces PGE_2_ levels. Mechanistically, we find that KLF11 performs these functions by binding to distinct sites in the *cPLA2*α promoter and recruiting chromatin silencer complexes. Interestingly, this effect can be blocked by the EGFR-AKT-mediated phosphorylation of Thr-56, a key regulatory residue within the KLF11-R1 domain also known as Sin3a interacting domain. Therefore, to our knowledge, the current study clearly outlines for the first time the role of any KLF protein in the down-regulation of *cPLA2*α and a consequent decrease in PGE_2_ synthesis as well as the role of AKT in transcriptional regulation of *cPLA2*α. The biological importance of these novel biochemical pathways, through the down-regulation of PGE_2_ synthesis, may very likely have a pleiotropic impact on important cellular functions.

## EXPERIMENTAL PROCEDURES

##### Reagents and Cell Cultures

Unless specified, all reagents were from Sigma. Primary epithelial cell cultures (B-HGD (36), h-TERT immortalized BAR-1 cell line (Dr. Jerry Shay, University of Texas Southwestern, Dallas, TX (37)), Chinese hamster ovary cells (ATCC, grown in F12 medium) and adenocarcinoma cell lines FLO, SEG-1, and SKGT-4 (Dr. David Beer (University of Michigan) and Dr. David Schrump (National Cancer Institute, Bethesda) (38) were used as previously published.

##### Proliferation Assays

Cell proliferation was assessed using MTS assay (Roche Applied Science) and confirmed with anti-BrdUrd antibody (Invitrogen) to detect BrdUrd (Roche Applied Science) incorporation in Hoechst 33742 (Invitrogen) counterstained cells. For transfection and adenoviral infection experiments, cells were green fluorescent protein co-transfected/co-infected and four different high-power fields were counted using a confocal microscope to determine the proliferation rates of green fluorescent protein-positive cells (39).

##### Cytosolic Phospholipase A_2_ Promotor Reporter Constructs, Luciferase Assay, PCR, and cPLA2 Enzyme Activity

The *cPLA2*α promoter reporter construct containing the sequence from −1200 to +150 relative to transcription start site in pGL2 (Promega, Madison, WI) was kindly provided by Dr. Mark Cowan (University of Maryland, Baltimore, MD). Promoter reporter deletion constructs of *cPLA2*α were cloned into the SacI and NheI sites of the pGL2 luciferase reporter vector (Promega) and verified by sequencing at the Mayo Clinic Molecular Biology Core Facility. Using the *cPLA2*α promoter reporter construct containing the sequence from −300 to +150 base pairs, the GC-rich site, ggagaccagcccacattttagcccctcctactc, was mutated to ggagaccagttcacattttagcccctcctactc (core SDM1) or ggagaccagcccacattttagcttctcctactc (core SDM2) via the QuikChange site-directed mutagenesis kit (Stratagene, La Jolla, CA) as recommended by the manufacturer.

Unless specified, for all transfection experiments, 50–60% confluent cells in six-well plates were treated with 1 ml of Opti-MEM (Invitrogen) containing 12 μl of Lipofectamine (Invitrogen) and 1.2 μg of total DNA. 48 h later, luciferase activity was measured after normalization (40, 41).

The *cPLA2*α mRNA expression was examined using semi-quantitative PCR (primer sequence and PCR conditions are outlined in supplemental Fig. S1) and *cPLA2*α enzyme activity was assessed using the *cPLA2* assay kit (Cayman Chemical, Ann Arbor, MI).

##### KLF11 Plasmids and Recombinant Adenovirus

Standard molecular biology techniques were used to clone full-length KLF11, as well as KLF11 deletions containing isolated R1 (amino acids 24–41), R2 (amino acids 151–162), or R3 (amino acids 273–351) fused to the C-terminal domain containing zinc fingers into pcDNA3.1/His (Invitrogen) as previously described (40, 41). Using full-length KLF11 in pcDNA3.1/His as a template, an extensive library of KLF11 constructs were generated to mutate serine and threonine phosphorylation sites to a non-phosphorylatable alanine or a phosphomimetic aspartic acid, using the QuikChange site-directed mutagenesis kit (Stratagene). A KLF11 mutant ΔE29P/ΔA30P was also generated to examine the role of the co-repressor Sin3a in regulation of the *cPLA2*α promoter activity by KLF11 (41). All constructs were verified by sequencing. KLF11 (Ad5CMV.TIEG2) or empty vector (Ad5CMV) carrying recombinant adenovirus were generated in collaboration with the Gene Transfer Vector Core at the University of Iowa.

##### Purification of Phosphospecific Thr-56 KLF11 Antibody by Affinity Chromatography

A 12-mer KLF11 peptide spanning P-threonine 56 was synthesized, high pressure liquid chromatography purified, and conjugated to keyhole limpet hemocyanin by the Mayo Clinic Protein Core. Two rabbits were immunized with the peptide by Cocalico Biologicals (Reamstown, PA). The antiserum was affinity purified against the original phosphopeptide using the EZ^TM^ Sulfhydryl Reactive Antibody Production and Purification Kit according to the manufacturer's protocol (Pierce Biotechnology).

##### PGE_2_ Assay

PGE_2_ production was measured in the supernatants of cell cultures using an enzyme immune assay kit (Cayman Chemical) as previously described (28, 36). PGE_2_ levels were corrected for total protein measurements.

##### Co-transfection, Immunoprecipitation, and Western Blot Analysis

FLO cells (90% confluent in 6-well plates) were co-transfected with His-tagged KLF11 along with siRNA against AKT (AKT1, -2, and -3, ON-TARGETplus SMARTpool siRNA, Dharmacon) or scrambled siRNA using Lipofectamine 2000 (Invitrogen). Each well received 0.6 μg of DNA, 200 pmol of siRNA, 4 μl of Lipofectamine 2000, and 2250 μl of Opti-MEM I (Invitrogen). 48 h later, cell lysate was precleared with Protein A/G-agarose bead slurry (Pierce) for 10 min at 4 °C. Immunoprecipitations were performed using Omni-probe D-8 antibody (Santa Cruz Biotechnology) overnight at 4 °C. The immunocomplexes were collected by incubating with Protein A/G-agarose beads for 1 h at 4 °C followed by centrifugation and washing five times with lysis buffer. Western blot analysis was performed as described above using Omni-probe D-8 and anti-phospho-Thr-56-KLF11 antibodies.

##### Chromatin Immunoprecipitation Assay (ChIP)

FLO cells were transfected with full-length His-tagged KLF11 expression constructs or a control vector as described above. 36 h after transfection, ChIP (EZ-ChIP kit, Upstate Biotechnology, Lake Placid, NY) was performed as previously described (42). PCR products were examined on a 2% agarose gel for enrichment of *cPLA2*α promoter in KLF11-transfected cells compared with controls cells using the following primers: forward, 5′-caatcttggctcactgcaagctct-3′ and reverse, 5′-tcacgcctgtaatcccagcacttt-3′.

##### Gel Shift Assays

To generate GST fusion KLF11-zinc finger protein, BL21 bacteria were induced with isopropyl 1-thio-d-galactopyranoside and recombinant fusion proteins were purified using GST-Sepharose beads (Amersham Biosciences) as previously described (41). Gel shift assay was performed using the digoxigenin gel shift kit (second generation) as per manufacturer's directions.

##### Whole Cell Extracts and Western Blot Analysis

Concomitant with transfection for the promoter reporter assays as well as independent experiments under identical experimental conditions, cell lysates were examined using standard Western blot techniques to determine protein expression levels (28, 36). Antibodies used included pan-AKT (C67E7, Cell Signaling Technologies, Danvers, MA), phospho-AKT (Ser473, D9E, Cell Signaling), His-A (Santa Cruz), and phospho-Thr-56-KLF11.

##### EGFR-AKT Plasmids and Pharmacological Inhibitors

Constitutively active and dominant-negative AKT constructs were kindly provided by Sushoven Guha (M.D. Anderson Cancer Center, Houston, TX). Standard molecular biology techniques were used to clone vErbB into pcDNA3.1/His (Invitrogen) as previously described (43). All constructs were verified by sequencing. The following inhibitors were purchased from EMD Chemicals, Inc. (Gibbstown, NJ) and used in the concentrations listed: 10 μm PD168393 (EGFR blocker), 100 μm LY294002 (phosphatidylinositol 3-kinase inhibitor), and 1 μm KP372–1 (AKT inhibitor).

##### Statistical Analysis

Results are expressed as mean ± S.E. Each experiment was repeated in triplicate at least three times. An overall F-test of treatment mean equality and Bonferroni method of multiple comparisons (*t* tests) were used. Statistical analysis was performed using SAS software (version 6.12; SAS Institute, Cary, NC). All statistical tests were two-sided.

## RESULTS

##### KLF11 Represses the Rate-limiting Enzyme cPLA2α and Down-regulates PGE_2_ Synthesis

**FIGURE 1:**
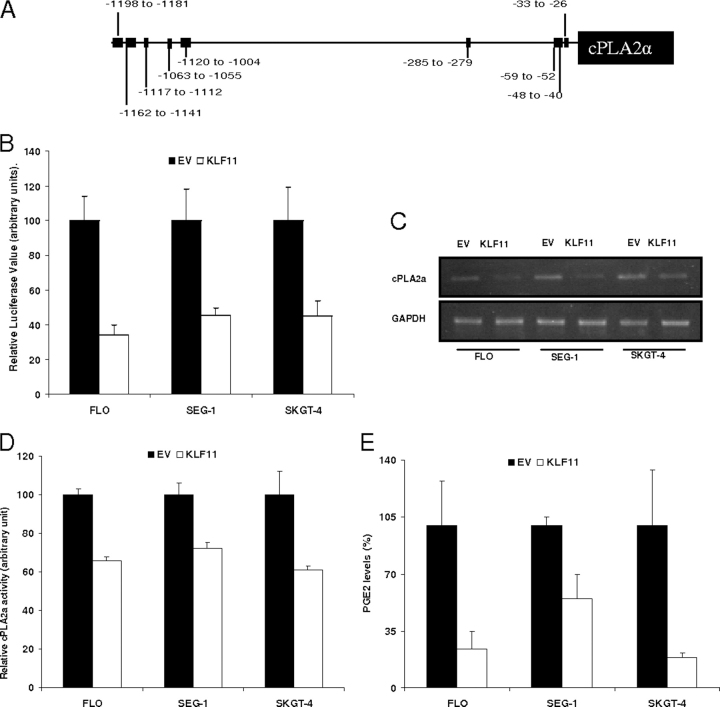
**KLF11 represses the rate-limiting enzyme *cPLA2*α and down-regulates PGE_2_ synthesis.***A,* the promoter region of *cPLA2*α contains several GC-rich, putative KLF11 binding sites. *B*, esophageal adenocarcinoma (FLO, SEG-1, and SKGT-4) cell lines were co-transfected with a *cPLA2*α promoter luciferase reporter construct (−1200 to +150 relative to transcription start site) along with full-length KLF11 or the EV construct for 48 h. Luciferase levels normalized to lysate protein concentrations show that compared with EV, co-transfection with KLF11 decreased *cPLA2*α promoter activity in FLO by 66 ± 3.3%, SEG-1 by 54.4 ± 5.8%, and SKGT-4 by 45 ± 9% (*p* < 0.05). As a negative control, the cyclin B1 promoter was used where KLF11 failed to decrease the promoter activity (data shown in supplemental Fig. S1). *C* and *D,* compared with empty vector, adenoviral infection of cells for 48 h with KLF11 decreased *cPLA2*α expression in all three cell lines and significantly reduced *cPLA2*α activity in FLO cells by 34% (474 ± 13 *versus* 319 ± 2.4 arbitrary units (AU), *p* < 0.05), in SEG-1 cells by 28% (572 ± 34 *versus* 407 ± 3 AU, *p* < 0.05), and in SKGT-4 by 39% (509 ± 12.8 *versus* 309 ± 1.6 AU, *p* < 0.05). *E,* compared with empty vector, adenoviral infection for 48 h with KLF11 also significantly reduced PGE_2_ production in FLO cells by 78% (86.7 ± 22 *versus* 19.4 ± 8.9 pg, *p* < 0.05), in SEG-1 cells by 46% (19.7 ± 9 *versus* 10.7 ± 3.1 pg, *p* < 0.05), and in SKGT-4 by 81% (100.4 ± 35 *versus* 19 ± 2.7 pg, *p* < 0.05). *GAPDH*, glyceraldehyde-3-phosphate dehydrogenase.

As mentioned, *cPLA2*α is a rate-limiting enzyme involved in the regulation of PGE_2_ synthesis (3, 11, 44, 45) and because PGE_2_ plays a direct role in important biochemical processes and pathological states (13, 15, 23, 28, 31, 36, 46, 47, 48, 49, 50), a tight regulation of this pathway is of paramount importance for homeostasis and diseases. Therefore, we began our studies on the role of KLF proteins in the regulation of PGE_2_ by applying bioinformatics promoter sequence analysis to look for KLF binding sites within established enzyme promoters from the PGE_2_ pathway (PubMed). Based upon this screening, *cPLA2*α became an interesting potential KLF11 target because the *cPLA2*α promoter region (Fig. 1*A*) contains several previously described GC-rich binding sites for this transcription factor (35). These bioinformatics-based predictions prompted us to ask first, whether KLF11 can regulate the *cPLA2*α-PGE_2_ pathway. To address this question, we co-transfected cells with a *cPLA2*α reporter construct (from −1200 to +150) either with full-length KLF11 or empty vector. Compared with empty vector, co-transfection with KLF11 decreased *cPLA2*α promoter activity in three different cancer cell lines, namely in FLO cells (66 ± 3.3%), SEG-1 (54.4 ± 5.8%), and SKGT-4 (by 45 ± 9%) (*p* < 0.05, Fig. 1*B*). Importantly, this KLF11-mediated inhibition of *cPLA2*α promoter activity was associated with decreased *cPLA2*α mRNA expression in all three cell lines (Fig. 1*C*). Congruent with this data, there was a 34% reduction in *cPLA2*α enzyme activity in FLO cells infected with the KLF11 adenovirus (474 ± 13 *versus* 319 ± 2.4% KLF11 Adv, compared with empty Adv controls, *p* < 0.05). Similarly, a down-regulation of *cPLA2*α enzyme activity was confirmed in KLF11-infected SEG-1 as well as SKGT-4 cells (Fig. 1*D*). Furthermore, KLF11 adenoviral infection of FLO, SEG-1, and SKGT-4 cells led to a 45–81% reduction in PGE_2_ production, the final catalytic product of *cPLA2*α enzyme activity (Fig. 1*E*). Collectively, these results demonstrate that KLF11 represses both *cPLA2*α promoter and enzyme activity although it decreases PGE_2_ production.

##### KLF11-mediated Down-regulation of cPLA2α-PGE_2_ Inhibits Cell Proliferation

The experiments thus far described demonstrate that KLF11 down-regulates *cPLA2*α-PGE_2_, although it does not indicate whether these changes impact on any cell function expected from this pathway. Thus, to test the premise that KLF11-mediated down-regulation of the *cPLA2*α-PGE_2_ pathway has any biological implications, we took a stepwise approach to examine its effects on cell proliferation. We first treated epithelial cells with either *cPLA2*α inhibitor or the catalytic products of *cPLA2*α, namely arachidonic acid or PGE_2_ to examine the relevance of *cPLA2*α-PGE_2_ pathway to the cell biological process of proliferation. Next, we overexpressed KLF11 using AdvKLF11 infection of cells to assess if KLF11 can regulate cell proliferation. Finally, we examined if the catalytic products of *cPLA2*α abrogates the effect of KLF11 on cell proliferation.

**FIGURE 2:**
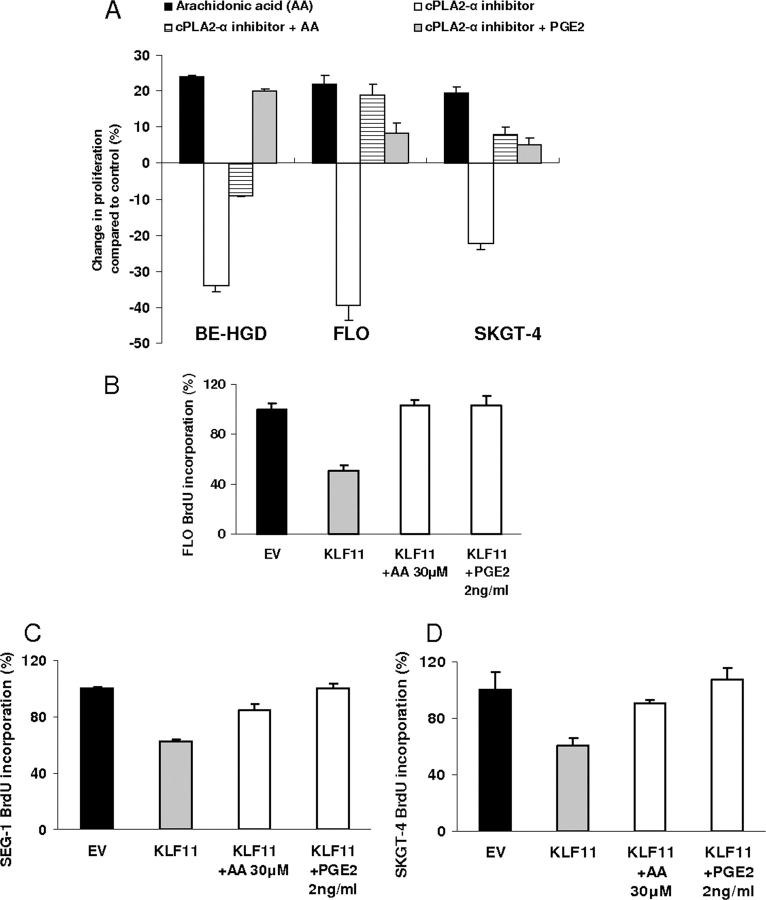
**KLF11-mediated down-regulation of *cPLA2*α-PGE_2_ inhibit cell proliferation.***A,* to test the cell biological significance of the *cPLA2*α-PGE_2_ pathway, FLO cells were treated for 48 h with 30 μm arachidonic acid, a catalytic product of *cPLA2*α, and increased proliferation by 22 ± 2.6%, whereas AACOCF3, a *cPLA2*α inhibitor, decreased proliferation by 39.5 ± 0.4% compared with control (BrdUrd incorporation, *p* < 0.05). The effect of AACOCF3α on FLO cell proliferation was reversed by the catalytic product of *cPLA2*α namely arachidonic acid and PGE_2_. Similar results were noted using other esophageal cancer cells (SKGT-4 and BE-HGD). These findings support that the effect of *cPLA2*α on cell proliferation is mediated by the release of arachidonic acid and production of PGE_2_. *B–D,* compared with EV, adenoviral infection of FLO (multiplicity of infection 30), SEG-1 (multiplicity of infection 100), and SKGT-4 (multiplicity of infection 100) cells for 48 h with KLF11 significantly (*p* < 0.05) reduced BrdUrd incorporation in cells that were treated with vehicle (49.5 ± 4.7, 38.5 ± 1.6, and 40 ± 5.9%, respectively for FLO, SEG-1 cells, and SKGT-4, *p* < 0.05), however, this growth inhibitory effect of KLF11 was abrogated in the cells that were treated with 30 μm arachidonic acid (AA, a catalytic product of *cPLA2*α and substrate of PGE_2_) or 2 ng/ml of PGE_2_ suggesting that the growth inhibitory effect of KLF11 is mediated via down-regulation of *cPLA2*α-PGE2 pathway.

First, to test the effects of the *cPLA2*α-PGE_2_ pathway on cellular proliferation, epithelial cells (BE-HGD, FLO, and SKGT-4) were treated with either the catalytic product of *cPLA2*α (30 μm arachidonic acid) or an inhibitor of *cPLA2*α (40 μm AACOCF3α). In FLO cells, compared with control, arachidonic acid increased proliferation by 22 ± 2.6% (*p* < 0.05). In contrast, AACOCF3α decreased cell proliferation by 39.5 ± 0.4% (*p* < 0.05) (Fig. 2*A*). Interestingly, in the presence of either arachidonic acid (PGE_2_ substrate) or PGE_2_ (final catalytic product of *cPLA2*α), the inhibitor of the *cPLA2*α enzyme failed to decrease cell proliferation. Therefore, this evidence supports the idea that the effects of *cPLA2*α on cell proliferation are mediated via arachidonic acid release and PGE_2_ production. Because KLF11 not only represses the *cPLA2*α promoter but also decreases PGE_2_ production, it is likely that the growth regulatory function of this transcription factor is mediated via the *cPLA2*α-PGE_2_ pathway under these circumstances. Consequently, we measured proliferation of epithelial cells (naturally expressing low KLF11 levels) that were transduced with KLF11, and treated for 48 h with vehicle, 30 μm arachidonic acid, or 2 ng/ml of PGE_2_. Under these conditions, infection with KLF11 decreased the BrdUrd-positive cells by up to 50% in vehicle-treated cells. This antiproliferative effect of KLF11 was completely abrogated in cells treated with either arachidonic acid or PGE_2_ (Fig. 2, *B–D*). Therefore, these results demonstrate that the antiproliferative effect of KLF11 is indeed mediated via down-regulation of the *cPLA2*α-PGE_2_ pathway in epithelial cells.

##### Silencing of cPLA2α Requires Defined KLF11-mediated Promoter Site Recognition and Chromatin Remodeling

**FIGURE 3:**
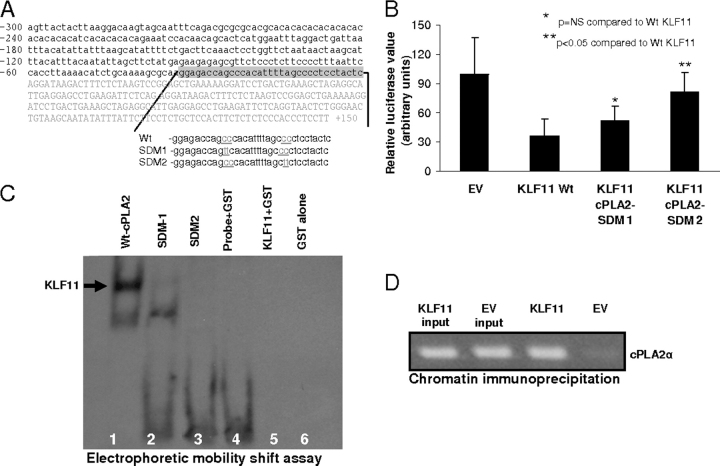
**KLF11-mediated regulation of *cPLA2*α requires defined promoter site recognition and KLF11 binds to *cPLA2*α promoter *in vivo*.***A,* outlining of site-directed mutagenesis in GC-rich areas of the *cPLA2*α promoter reporter construct. *B*, compared with control, KLF11 was able to repress the *cPLA2*α-WT promoter but failed to repress the *cPLA2*α promoter that had cc to tt mutations in the distal GC-rich site (*cPLA2*α-WT (36.29 ± 17.7%) *versus cPLA2*α-SDM2 (80.8 ± 20.9%), *p* < 0.05). The mutations in the more proximal GC-rich site (SDM1) only partially relived the KLF11-dependent repression (52 ± 14.7%), which was not significantly different compared with *cPLA2*α-WT. *C,* electrophoretic mobility shift assay shows that binding of the KLF11-GST recombinant protein and digoxigenin-labeled fragment of the *cPLA2*α core promoter sequence was partially disrupted with SDM-1 mutations (*lane 2*) and completely disrupted by SDM-2 mutations (*lane 3*) in the GC-rich sequence of the *cPLA2*α promoter. *Lane 1* represents the fragments containing the wild-type *cPLA2*α core promoter sequence (*Wt-cPLA2*α) and *lanes 4–6* are negative controls. *D*, ChIP assay using FLO cell lysates shows that the *cis*-regulatory *cPLA2*α promoter sequence is enriched in immunoprecipitated samples from cells infected with KLF11-carrying adenovirus and absent in EV control-infected cells demonstrating that KLF11 can bind to the promoter of *cPLA2*α endogenously.

To better understand the molecular mechanisms involved in regulation of the *cPLA2*α promoter by KLF11, we used an extensive battery of approaches, including bioinformatics, deletions, and site-directed mutagenesis, as well as electrophoretic mobility shift and ChIP assays. For these experiments, to map the KLF binding sites within the *cPLA2*α promoter, we co-transfected KLF11 with either wild-type core *cPLA2*α promoter reporter (*cPLA2*α-WT from −300 to +150 relative to its transcription start site, which behaves similar to a 1200-bp fragment in its responsiveness to KLF11-dependent repression) or the same construct carrying CC to TT mutations at one of the two GC-rich sites, referred here as SDM1 or SDM2 (Fig. 3*A*). Interestingly, KLF11 is unable to repress the *cPLA2*α promoter mutated at the distal GC-rich SDM2 site (*cPLA2*α-WT (36.2 ± 17%) *versus* SDM2 (80.8 ± 20.9%), *p* < 0.05, Fig. 3*B*). Mutation of the proximal GC-rich SDM1 site did not significantly alter KLF11-dependent repression (*cPLA2*α-WT (36.2 ± 17%) *versus* SDM1 (52 ± 14.7%), *p* > 0.05, Fig. 3*B*). Binding of KLF11 to these sites was determined by electrophoretic mobility shift assay. These experiments revealed that binding of KLF11 to the *cPLA2*α promoter was completely disrupted with SDM2 mutation, which is required for promoter silencing via KLF11 (Fig. 3*C*). To address whether KLF11 binds to the endogenous *cPLA2*α promoter, we used chromatin immunoprecipitation assays. The results of these experiments demonstrate that the *cPLA2*α promoter can be occupied *in vivo* following overexpression of KLF11 (Fig. 3*D*). Together, these mechanistic findings demonstrate that at the *cis*-regulatory level, KLF11 binds to and represses the *cPLA2*α promoter via a distinct GC-rich site. Furthermore, KLF11 not only occupies the *cPLA2*α promoter *in vivo* but it also behaves as a *bona fide* repressor of this gene.

**FIGURE 4:**
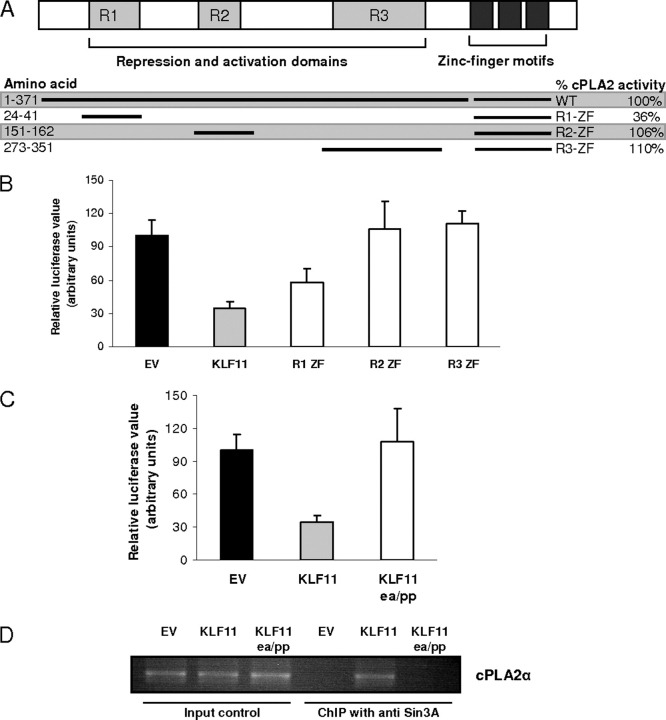
**R1 repressor domain of KLF11 is critical in the *cPLA2*α promoter repression via binding and function of the Sin3a-HDAC chromatin remodeling complex.***A,* the *top panel* shows the outline of repressor and DNA binding domains of the KLF11 protein. The *lower panel* is a summary as described in the legend to Fig. 3*B. B,* FLO cells were co-transfected with the *cPLA2*α promoter reporter construct along with either empty vector or full-length KLF11 or KLF11 deletions containing distinct regulatory domains. Compared with empty vector control, both full-length and R1-ZF KLF11 significantly repressed *cPLA2*α promoter activity (65.6 ± 12 and 42.4 ± 9.8%, *p* < 0.05) but R2-ZF and R3-ZF failed to repress the *cPLA2*α promoter activity. *C,* in FLO cells, compared with control, wild-type KLF11 repressed the *cPLA2*α promoter activity by 65.6 ± 12% (*p* < 0.05), the ΔE29P/ΔA30P-KLF11 (the mutant to disrupt Sin3a-HDAC binding) completely abolished *cPLA2*α repression by KLF11. *D*, ChIP assay using FLO cell lysates shows that the *cis*-regulatory *cPLA2*α promoter sequence is enriched in anti-Sin3a antibody (SC-994) immunoprecipitated samples from cells infected with adenovirus carrying wild-type KLF11 (*fifth lane* from the *left*) but absent in adenovirus carrying the ΔE29P/ΔA30P-KLF11 mutation (*sixth lane* from the *left*) demonstrating that the KLF11-mediated recruitment of Sin3a to the *cPLA2*α promoter can be abrogated by the ΔE29P/ΔA30P mutation in KLF11. The input controls are in the *first three lanes* on the *left* and similar results were noted in SEG-1 cells (data not shown). Together, the data support that the R1 domain of KLF11 is critical in repression of the *cPLA2*α promoter and this repression is Sin3a-HDAC-dependent.

Recent investigations have demonstrated that KLF11 functions with multiple chromatin remodelers that bind independently to its repressor domains R1, R2, and R3, and the zinc fingers (40, 41). In addition, each KLF11 domain appears to function via different chromatin remodeling machines. Therefore, we investigated which mechanism KLF11 employs for repression of the *cPLA2*α promoter. Consequently, in an unbiased approach, we initially investigated which of these functional domains, known to bind to different chromatin remodeling machines, are necessary for regulation of *cPLA2*α. We used several KLF11 constructs including full-length KLF11, as well as KLF11 deletions containing its individual chromatin-binding regulatory domains fused to the C-terminal domain containing zinc fingers (R1-ZF, R2-ZF, and R3-ZF) (Fig. 4*A*, *upper panel*). Co-transfection of FLO cells with the *cPLA2*α promoter reporter construct and these various constructs revealed that compared with empty vector control, R1-ZF repressed *cPLA2*α promoter activity by 42.4 ± 9.8%, a value not statistically different from the repression exerted by full-length KLF11 protein (65.6 ± 12%, *p* > 0.05). In contrast, R2-ZF and R3-ZF failed to repress *cPLA2*α promoter activity (Fig. 4*A*, *lower panel*, and *B*). These findings illustrate that the KLF11 R1 domain is functionally important and sufficient in the regulation of the *cPLA2*α promoter. From our results, we also conclude that the chromatin remodeling machines bound by KLF11 via the R2, R3, and zinc finger domain are unlikely to be involved in this process. Because the R1 domain contains sequences that mediate interaction with the chromatin co-repressor complex, Sin3-HDAC (40, 41), this information led us to experimentally confirm whether this corepressor system is directly involved in this repression effect. We then compared the regulatory potential of the KLF11 construct that carries a mutation that interferes with Sin3a binding to KLF11 (ΔE29P/ΔA30P-KLF11) (41). As shown in Fig. 4*C*, this mutation abolished *cPLA2*α repression by KLF11. Based upon these findings, we speculated that the E29P/A30P mutation in the R1 domain of KLF11 interferes with Sin3-HDAC recruitment by KLF11 and therefore, KLF11 is not able to repress the *cPLA2*α promoter. To test this idea, we infected FLO as well as SEG-1 cells with adenovirus carrying a control empty parental vector, wild-type KLF11, or KLF11 with the ΔE29P/ΔA30P mutation. As shown in Fig. 4*D*, ChIP assays with an anti-Sin3a antibody demonstrate that whereas Sin3a occupies the *cPLA2*α promoter in cells infected with wild-type KLF11, the recruitment of this corepressor was abrogated by the ΔE29P/ΔA30P-KLF11 mutant. Together, these findings led us to conclude that the R1 domain is critical in repression of the *cPLA2*α promoter and this phenomenon depends upon the binding and function of the Sin3a-HDAC complex. Furthermore, contrary to repression by histone methylation (*e.g.* polycomb), which is long lived (51, 52), histone acetylation by Sin3a/HDAC is meant to be short lived and susceptible to antagonism by signaling (53, 54, 55). Therefore, in the following paragraph we describe experiments that explore the idea of a more dynamic regulation of *cPLA2*α by signaling pathways that could potentially affect KLF function.

##### Membrane to Nucleus Signaling-dependent Post-translational Changes in KLF11 Can Modulate KLF1-dependent cPLA2α Regulation

**FIGURE 5:**
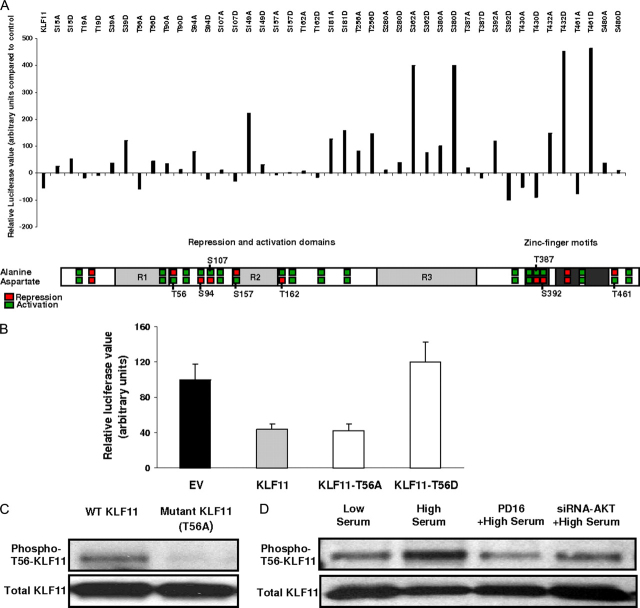
**Post-translational modification of threonine at position 56 in KLF11, a target of phosphorylation by AKT, is crucial in KLF11-mediated repression of the *cPLA2*α promoter.***A,* Chinese hamster ovary cells were co-transfected with the *cPLA2*α promoter reporter construct along with either EV or KLF11 constructs from a library of mutant KLF11 proteins where serines and threonines were replaced with either alanines or aspartic acids as indicated. The eight phosphomimetic and non-phosphorylatable KLF11 mutants with opposing effects on *cPLA2*α promoter activity are displayed along with a KLF11 protein domain outline. *B,* FLO cells co-transfected with the *cPLA2*α promoter reporter construct along with either empty vector or a phosphomimetic (T56D) or non-phosphorylatable (T56A) KLF11 mutant in the R1 domain (in close proximity of its Sin3a binding site) shows that at 48 h, compared with control, wild-type KLF11 repressed the *cPLA2*α promoter activity to 43 ± 6% but the phosphomimetic T56D-KLF11 mutant resulted in a complete release of *cPLA2*α promoter repression by KLF11 (120 ± 23%). The repression of *cPLA2*α persisted with the T56A-KLF11 mutant (42 ± 7%). *C*, lysates from Chinese hamster ovary cells transfected with wild-type KLF11 or T56A mutant KLF11 after immunoprecipitation of His-tagged KLF11 followed by Western blot with phospho-Thr-56-KLF11 antibody shows the specificity of this antibody as it does not bind to non-phosphorylatable T56A-KLF11. *D,* KLF11-transfected FLO cells were treated with either scrambled siRNA + vehicle or siRNA against AKT (AKT-1, -2, and -3) transfection or PD168393 (EGFR blocker) to inhibit AKT. 24 h later cells were either maintained in 5% FBS (low serum) or given a 90-min pulse of high serum medium (10% FBS to activate EGFR-AKT pathway). After immunoprecipitating His-tagged KLF11 protein, resolving by 10% SDS-PAGE, and immunoblotting with anti-phospho-Thr-56-KLF11 and total KLF11, as a loading control, we found that the high serum pulse that activates AKT (data shown in supplemental Fig. S6, 2 and 3) results in phosphorylation of Thr-56 in KLF11 and that the siRNA against AKT, as well as PD168393 to inhibit AKT, markedly reduced the phosphorylation of this site.

To gain more robust biochemical insight into the KLF11-mediated *cPLA2*α repression, we used an unbiased molecular screening to search and define potential post-translational modifications in KLF11 that could be a target of conspicuous signaling cascades. Guided by bioinformatics-based analyses (“Experimental Procedures”), we performed extensive mutagenesis to replace the serines and threonines that were candidates for modification by intracellular signaling kinases with either an alanine (non-phosphorylatable mutation) or aspartic acid (phosphomimetic mutation). The functional impact of these mutations on the ability of KLF11 to repress the *cPLA2*α promoter was examined (Fig. 5*A*). This screening demonstrates that the KLF11-mediated repression of the *cPLA2*α promoter could be modulated by various signaling-induced post-translational modifications.

Because this data raised the question of whether any of these modifications indeed occur or have a function *in vivo*, we elected to study phosphorylation of one of these sites, KLF11-Thr-56, as a model. This choice was based on computer-assisted predictions showing that KLF11-Thr-56, which lies within the R1 domain adjacent to the Sin3a-binding domain, is a target for AKT phosphorylation, and our *in silico* KLF11 protein analysis suggests that changes in charge and/or structure generated by addition of the phosphate to the threonine may have functional consequences. Moreover, to our knowledge the effect of AKT on *cPLA2*α, the rate-limiting enzyme of the PGE_2_ biosynthesis pathway has not been previously reported. This choice was further supported by the finding that a phosphomimetic mutation at Thr-56 (Thr to Asp) results in release of *cPLA2*α promoter repression by KLF11 but the repression persists with an alanine, non-phosphorylatable, mutation at Thr-56 (EV (1 ± 0.18) and KLF11-WT (0.43 ± 0.06) *versus* KLF11-T56D (1.2 ± 0.23) and KLF11-T56A (0.42 ± 0.07); Fig. 5*B*). This data, together with the fact that the EGFR-AKT pathway plays a significant role in both biology and diseases (56, 57, 58, 59, 60, 61, 62), led us to investigate whether modulating this signaling cascade alters the phosphorylation of KLF11-Thr-56. To facilitate these studies, we developed a phospho-Thr-56-KLF11 antibody. The specificity of this antibody was confirmed in epithelial cells transfected with either wild-type KLF11 or the non-phosphorylatable T56A-KLF11 mutant (Fig. 5*C*). FLO cells were transfected with KLF11 and treated with either the EGFR inhibitor PD168393 or AKT siRNA in low serum conditions (5% FBS). After 24 h, cells were either maintained in low serum or changed to high serum conditions (10% FBS) as a rough method to activate AKT (supplemental Fig. S6, 2 and 3). Notably, high serum exposure resulted in increased phosphorylation of Thr-56. More importantly, both pharmacological and genetic manipulations that inhibit EGFR-AKT decreased the phosphorylation of this site, even in the presence of a high serum pulse (Fig. 5*D*). Therefore, these experiments demonstrate that KLF11-Thr-56 is phosphorylated by AKT upon EGFR activation.

**FIGURE 6:**
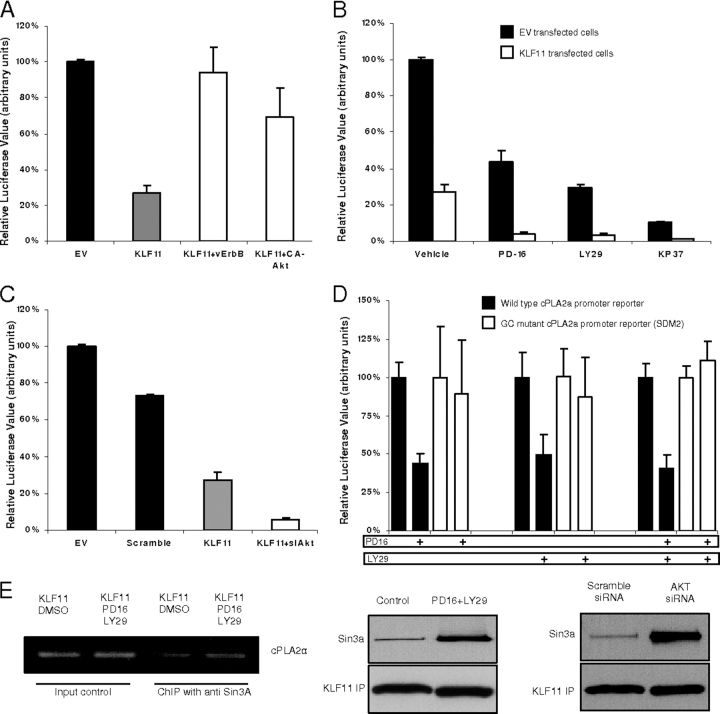
**EGFR-AKT signaling is involved in KLF11-mediated *cPLA2*α promoter repression.***A,* FLO cells were co-transfected with the *cPLA2*α promoter reporter construct along with either EV or KLF11, with or without vErbB (constitutively active EGFR), or CA-AKT. KLF11 overexpression reduced the *cPLA2*α promoter activity by 63 ± 4.3% (*p* < 0.05), however, this repression was released in the presence of vErbB and CA-AKT (6 ± 0.9 and 31 ± 7% repression, respectively, *p* < 0.05 compared with KLF11). *B,* FLO cells co-transfected with *cPLA2*α along with either EV or KLF11 were treated with either vehicle or the blockers of EGFR-AKT pathway (10 μm PD168393, 100 μm LY294002, or 1 μm KP372–1). 48 h later, KLF11 decreased the *cPLA2*α promoter activity in the presence of vehicle by 3.7-fold (100 ± 1.3 *versus* 27 ± 4.3%), with PD168393 by 10.7-fold (43 ± 6.5 *versus* 4 ± 1%, *p* < 0.05 compared with KLF11 with vehicle), with LY294002 by 9.5-fold (29.7 ± 1.6 *versus* 3 ± 0.9%, *p* < 0.05 compared with KLF11 with vehicle), and with KP372-1 by 10-fold (10 ± 1 *versus* 1 ± 0.05%, *p* < 0.05 compared with KLF11 with vehicle). *C,* FLO cells were co-transfected with *cPLA2*α along with either EV or KLF11 and siRNA against AKT or scramble RNA. Cells were maintained in 10% FBS for 48 h. KLF11 decreased *cPLA2*α promoter activity by 12-fold (73 ± 1 *versus* 6 ± 1%) in the presence of AKT siRNA compared with a 3.7-fold reduction (100 ± 1.3 *versus* 27 ± 4.3%) with scramble siRNA (*p* < 0.05). *D,* FLO cells transfected with either *cPLA2*α-WT promoter or *cPLA2*α-SDM2 (mutation in GC-rich sequence to which KLF11 binds). Cells were treated with vehicle, 10 μm PD168393, or 100 μm LY294002 or both PD168393 and LY294002 for 24 h in 10% FBS. PD168393 decreased the *cPLA2*α-WT promoter activity by 66% (100 ± 10 *versus* 44 ± 6%, *p* < 0.05) but had no significant effect on the *cPLA2*α-SDM2 promoter activity (100 ± 33 *versus* 89 ± 35%). A similar pattern was also noted with LY294002 alone or with both PD168393 and LY294002. *E,* KLF11-transfected FLO cells were treated with either vehicle or 10 μm PD168393 plus 100 μm LY294002 for 24 h in the presence of 10% FBS. Chromatin immunoprecipitation with anti-Sin3a antibody showed a slight increase in *cPLA2*α promoter enrichment in the blocker-treated group. To compliment this, FLO cells were either co-transfected with KLF11 and AKT siRNA (or scramble RNA control) or treated with 10 μm PD168393 plus 100 μm LY294002 (or vehicle control). Western blots after immunoprecipitation of His-tagged KLF11 followed by probing with the anti-Sin3a antibody shows that compared with control there was increased KLF11-Sin3a complexing in AKT blockers as well as AKT-siRNA-treated cells compared with the control. *DMSO*, dimethyl sulfoxide.

To define the functional effect of EGFR-AKT signaling on transcriptional repression of *cPLA2*α via KLF11, we used molecular and pharmacological activation or inhibition of the EGFR-AKT pathway (Fig. 6, *A–D*). FLO cells were co-transfected with *cPLA2*α along with either EV or KLF11, with or without vErbB or constitutively active-AKT (CA-AKT). The results of these experiments demonstrated that although KLF11 alone reduced the *cPLA2*α promoter activity by 63 ± 4.3%, this repression was markedly antagonized by vErbB and CA-AKT (6 ± 0.9 and 31 ± 7%, respectively, *p* < 0.05 compared with KLF11, Fig. 6*A*). To further substantiate these results, we co-transfected FLO cells with increasing CA-AKT to KLF11 ratios. Notably, increased CA-AKT released KLF11-dependent repression of the *cPLA2*α promoter (supplemental Fig. S6, 1). Conversely, blockers of the EGFR-AKT pathway (10 μm PD168393, 100 μm LY294002, or 1 μm KP372-1) enhanced KLF11-dependent repression of the *cPLA2*α promoter by 6–8-fold (*p* < 0.05 compared with blockers alone, Figs. 6*B* and supplemental S6, 2). Complementary studies using AKT siRNA displayed results similar to the ones obtained using small drug inhibitors (Figs. 6*C* and supplemental S6, 3). In conclusion, both pharmacological and genetic manipulation of EGFR-AKT signaling demonstrated a key role of this pathway in the regulation of KLF11-mediated repression of *cPLA2*α.

To define whether the EGFR-AKT pathway directly impacts on the KLF11-mediated regulation of this promoter at the appropriate *cis*-regulatory site, we repeated these experiments using either the WT or SDM2 mutant *cPLA2*α promoter. We find that inhibitors of EGFR-AKT signaling repress the wild-type *cPLA2*α promoter but not the SDM2 mutant (Fig. 6*D*). Thus, these results demonstrate that a functional KLF11 binding site is critical for AKT-mediated inhibition of *cPLA2*α transcription.

Finally, we examined whether EGFR-AKT inhibitors affect KLF11-dependent *cPLA2*α promoter repression via recruitment of Sin3a, using ChIP assay (Fig. 6*E*). These experiments showed that pharmacological blockers induce an enrichment of Sin3a on the *cPLA2*α promoter. These results were complemented by examining the effect of AKT inhibitors on KLF11-Sin3a complexing. For this purpose, KLF11-transfected FLO cells were treated with either the small drug blockers or AKT siRNA for co-immunoprecipitation studies. Both types of treatments increased the amount of Sin3a associated to KLF11. Thus, AKT inhibition increases the interaction between Sin3a and KLF11, allowing this protein to efficiently inhibit PGE_2_ synthesis by down-regulating the *cPLA2*α promoter via a distinct GC-rich binding site.

## DISCUSSION

The current study uncovers important mechanistic insights into novel molecular pathways for the regulation of prostaglandin synthesis by KLF proteins. As evident from the data presented in this study, KLF11 is the first Kruppel-like factor family member to be associated with the down-regulation of prostaglandin synthesis, particularly PGE_2_. The transcriptional silencing of *cPLA2*α, the key regulatory enzyme of PGE_2_, by KLF11 occurs via binding to distinct *cPLA2*α promoter elements as well as recruitment of Sin3a-HDAC activity. This activity, required for KLF11-mediated silencing, is antagonized by Thr-56 phosphorylation, which is mediated by the EGFR-AKT pathway. Consequently, these studies reveal previously unknown aspects of the biochemical regulation of prostaglandin biosynthesis at both the transcriptional level by KLF11 and at the level of post-translational changes in the KLF11 protein. Due to the involvement of prostaglandins in a myriad of cellular effects, both the biological and medical relevance of this information is significant.

**FIGURE 7:**
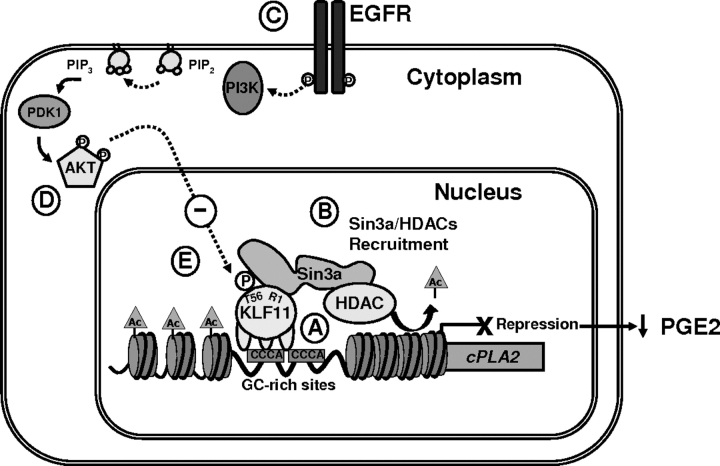
**Mechanistic model of KLF11-mediated tumor suppression and its antagonism by an oncogenic pathway.** KLF11 binds to the GC-rich consensus sequences in the promoter region of *cPLA2*α, the key rate-limiting enzyme of the oncogenic PGE2 cascade. *A*, KLF11 represses the *cPLA2*α promoter by recruiting the chromatin remodeling complex, Sin3a-HDAC (*B*). As a consequence, KLF11 behaves as a tumor suppressor in Barrett's epithelial cells, at least in part, by repression of the *cPLA2*α-PGE_2_ pathway. EGFR-AKT signaling, which is up-regulated in a subset of patients during carcinogenesis in Barrett's esophagus (*C*) phosphorylates threonine at position 56 in the R1 domain of KLF11 in the immediate vicinity of its Sin3a interacting domain (*D*), and reverses the KLF11-dependent repression of the *cPLA2*α promoter (*E*). Together, this model outlines mechanistic links, namely KLF11-SIN3a/HDAC-*cPLA2*α-PGE_2_ and EGFR/AKT-KLF11 (Thr-56 phosphorylation)-SIN3a/HDAC-*cPLA2*α-PGE_2_.

Although this study focuses on KLF11-mediated regulation of PGE_2_ synthesis via *cPLA2*α, interestingly, our own preliminary observations using TRANSFACT (data not shown) predicts that other members of this pathway may also be targets of the KLF proteins. This observation could therefore fuel further investigations into the exciting area of research involving the regulation of PGE_2_ synthesis. A model, as outlined in Fig. 7, integrates the novel signaling loop described here, which places KLF11 at the center of two novel pathways: the first, a KLF11-Sin3a/HDAC-*cPLA2*α pathway that down-regulates PGE_2_ synthesis and the second, an EGFR-AKT-KLF11-Sin3a/HDAC-*cPLA2*α pathway, where EGFR-AKT phosphorylates and functionally inactivates KLF11-mediated repression of *cPLA2*α. Concretely, KLF11 silences *cPLA2*α and down-regulates PGE_2_, unless it is antagonized via the EGFR-AKT pathway.

Mechanistically, at the transcriptional level, inhibition of the PGE_2_ pathway occurs via distinct *cPLA2*α promoter elements, and requires the recruitment of the chromatin remodeling, Sin3a-HDAC complex. Other potential chromatin remodeling proteins that could bind to the R2, R3, and zinc finger domains do not appear to be involved in KLF11-mediated *cPLA2*α promoter repression (Fig. 4). The sequences to which KLF11 binds in the *cPLA2*α promoter are GC-rich and match the consensus sites previously determined for this protein (35). The similarity among members of the SP/KLF family of proteins to recognize specific GC-rich sequences would predict that other members of this family may also act as regulators of this promoter. Indeed, it has been previously shown that Sp1 binds to the *cPLA2*α promoter in fibroblast cells from the lung, leading to its induction (63). These observations, when combined with that previously published by our laboratory (34, 64), postulate at least a minimum “ying-yang” mechanism, by which Sp1 would act as an activator and KLF11 as its silencer. Although both, Sp1 and KLF11, are ubiquitously expressed in all the tissues examined (data not shown), it would not be surprising if, similarly, other KLF proteins, with more restricted expression patterns, play a role in regulating prostaglandin biosynthesis, either in different cells or different tissues, and either as activators or repressors in a similar ying-yang manner.

This study also reveals an important biochemical finding as regards to the fine regulation of the KLF11-mediated silencing of *cPLA2*α. Using a completely unbiased approach involving a combination of bioinformatics and extensive site-directed mutagenesis (Fig. 5), we searched for evidence on the potential regulation of the KLF11-*cPLA2*α-PGE_2_ pathway by biochemically important signaling molecules. Surprisingly, this approach revealed that the EGFR-AKT pathway-mediated phosphorylation of Thr-56 alone can antagonize the repressive function of KLF11. This is a novel and potentially interesting observation because in previous investigations, using heterologous Gal4 proteins and promoter systems, we observed that phosphorylation of all of the 4 existing S*XX*P sites in KLF11 (some of them a slightly further from the R1 domain than Thr-56) by ERK-1 is required to disrupt KLF11-dependent silencing (43). Therefore, together this data demonstrates that phosphorylation of KLF11 within the region of its interaction with Sin3A is essential for neutralizing the function of KLF11 either by AKT or ERK-1 (Thr-56). However, ERK-1 requires additional sites for achieving the same function as AKT. Furthermore, this data also reveals unexpected yet vital information, that the EGFR can use two different intracellular pathways (ERK-2 *versus* AKT) to achieve the same function, namely inactivation of KLF11. Although, the phosphorylation required for KLF11 inactivation by these two pathways target partially the same region, the residues they phosphorylate are not identical. Because the data on the phosphorylation events that can regulate KLF family members and the functional consequences of these post-translational effects is scanty, the data from this study certainly contribute to expand our biochemical knowledge about KLF proteins.

Methodologically, our data demonstrates that the candidacy of phosphorylatable residues identified by bioinformatics outnumber the apparent candidates that can disrupt the function of this protein as identified by mutational and functional analysis, at least under basal conditions. This result is consistent with the predictive power of most bioinformatics algorithms. Therefore, based upon our results (bioinformatics *versus* mutational analysis (see Fig. 5), we predict that the existence of additional phosphorylation events, which may regulate R1-mediated repression, will be less likely using this methodology.

Another intriguing biochemical observation to discuss when considering the involvement of KLF11 in PGE_2_ synthesis is how this phenomenon has the potential to bring about variations in the PGE_2_ pathway. The potential variations could result from the existence of several functional KLF11 genetic variants. Some of these variants represent polymorphisms, whereas others are due to mutations. These genetic variants function differently than their normal counterparts and association studies have already linked these variants to juvenile diabetes (35). In fact, because these KLF11 variants cause disease, the gene has been recently renamed MODY VII (OMIN: ncbi.nlm.nih.gov). Of significant importance, these KLF11 genetic variants display variability in their binding to and the regulation of the insulin promoter in part due to the malfunction in their association with Sin3a. Notably, this is the domain that KLF11 uses to repress the *cPLA2*α promoter and is targeted by EGFR-AKT. Based upon these findings, future studies in our laboratory will seek to define whether potential variations in the regulation of PGE_2_ biosynthesis result from the existence of different KLF11 polymorphisms thus creating anomalies in the large number of functions associated with this prostaglandin. Therefore, because of the impaired binding of the KLF11 variant to Sin3a, the discovery of the mechanisms discussed in this article are of significant biochemical importance.

From the standpoint of *cPLA2*α-PGE_2_ pathway regulation, this study offers yet another novel finding. Although there is some evidence that AKT-dependent signaling can alter PGE_2_ synthesis by regulating COX-2 function, our study provides an additional mechanism where AKT, by causing post-translational changes in KLF11 (P-Thr-56), could alter the transcriptional regulation of *cPLA2*α.

In conclusion, together this study significantly expands our understanding of the role of KLF proteins on established and important biochemical pathways, such as prostaglandin biosynthesis. Krüppel-like factors are increasingly being recognized for their ability to govern important biological processes that are involved in mammalian development, differentiation, survival, and aging (25, 26, 27). Thus, it is likely that KLF-prostaglandin pathways are important regulators of these phenomena.

## Footnotes

2 The abbreviations used are:

PGE_2_: prostaglandin E_2_
COX-2: cyclooxygenase-2
GST: glutathione *S*-transferase
CA-AKT: constitutively active-AKT
BrdUrd: bromodeoxyuridine
EGFR: epidermal growth factor receptor
siRNA: small interfering RNA
ChIP: chromatin immunoprecipitation
WT: wild-type
FBS: fetal bovine serum
ERK: extracellular signal-regulated kinase
EV: empty vector.
