# Supplementary material for: Distinct Role of Kruppel-like Factor 11 in the Regulation of Prostaglandin E2 Biosynthesis
Source: J Biol Chem. 2020 Oct 23;285(15):11433–44. doi: 10.1074/jbc.M109.077065 (PMC2857022; doi:10.1074/jbc.M109.077065)
Supplement: Supplementary file 1 [file mmc1.pdf]

FIGURE 1S

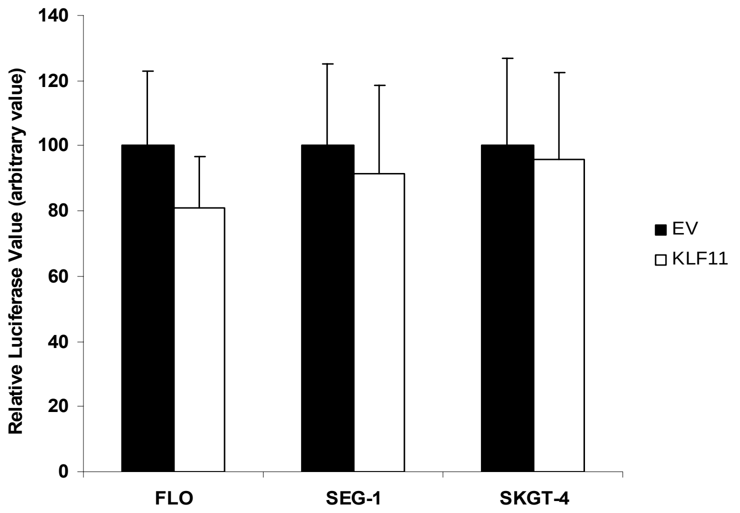

FIGURE 6S1

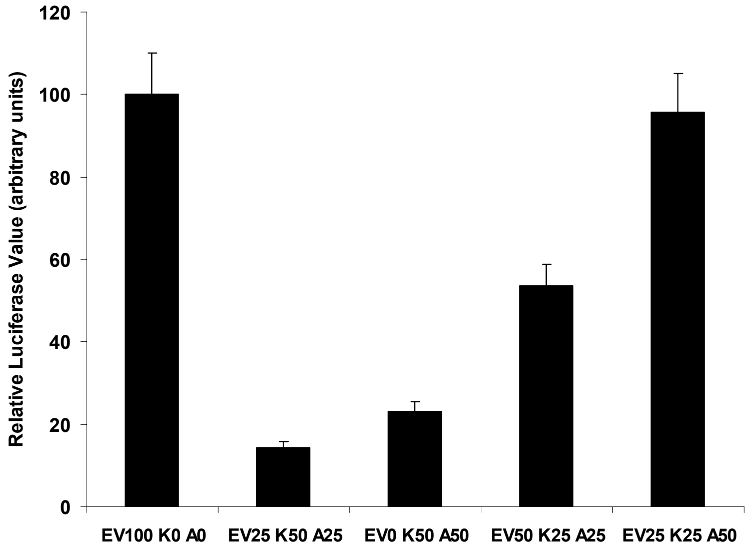

FIGURE 6S2

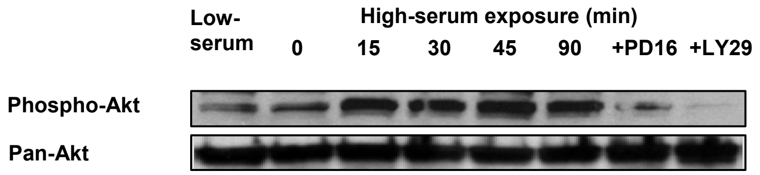

FIGURE 6S3

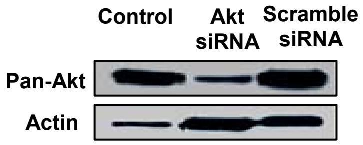

FIGURE 6S4

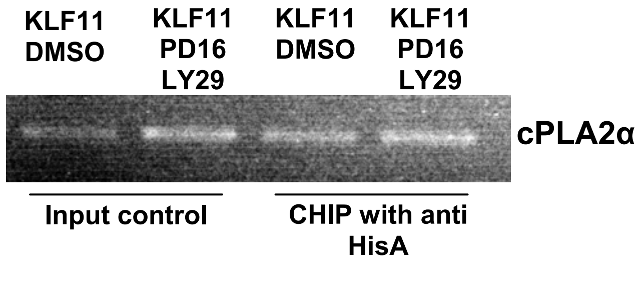

Supplementary Figure Legends

Figure 1S: KLF11 fail to decrease the Cyclin B1 promoter activity: Esophageal adenocarcinoma (FLO, SEG-1, SKGT-4) cell lines were co-transfected with a *cPLA2α* promoter luciferase reporter construct (-1200 to +150 relative to TSS) along with full-length KLF11 or EV construct for 48 hours. Luciferase levels normalized to lysate protein concentrations show that compared to empty vector, the co-transfection with KLF11 did not change the *cPLA2α* promoter activity in FLO (81±15%), SEG-1 (91±27%) or SKGT-4 (95±26%) cells ($p>0.05$).

Figure 6S1: Sustained activation of AKT can overcome the effect of KLF11 on *cPLA2α* promoter activity: FLO cells were co-transfected with indicated ratios of EV, KLF11 (K), and CA-AKT (A) and maintained in media containing 1% FBS for 48 hours. Increasing Ca-AKT, in presence of fixed KLF11, relieved cPLA2α promoter repression. The second (EV25K50A25) and third (EV0K50A50) lanes have high, but similar, amounts of the KLF11 expression construct (5ug in each) and the 4th as well as 5th lanes have low, but similar, amounts (2.5 ug each). Given that by increasing Ca-AKT in presence of fixed KLF11, there was a release in cPLA2 promoter repression ($p<0.05$ when lane 5 is compared to lane 6), it appears that KLF11 dependent repression of cPLA2 promoter is reversible but requires significant and sustained activation of AKT to overcome the effect of KLF11.

Figure 6S2: High serum pulse induces phospho AKT levels, which can be prevented by pharmacological inhibitors of EGFR-AKT: KLF11 transfected FLO cells were treated with either the EGFR inhibitor (10 µM PD168393) or PI-3K inhibitor (100 µM LY294002). After 24-hours, cells were either maintained in low serum (5% FBS) or changed to high serum conditions (10% FBS). Western blot after immunoprecipitating total AKT (Cell Signaling, #4691) and probing with phospho specific AKT antibody (Cell Signaling, #4060) shows that a 90 minute pulse of high serum activates AKT but this activation is prevented by EGFR-AKT cascade inhibitors (PD168393 and LY294002).

Figure 6S3: SiRNA against AKT can downregulate AKT levels: FLO cells were co-transfected with KLF11 along with either AKT siRNA or Scrambled siRNA (ON-TARGETplus SMART pool, Dharmacon) using TransIT-TKO tranfection reagent (Mirus) according to manufacturer's protocol and maintained in 10% FBS. After 48-hours, Western blot shows that compared to transfection reagent control or scrambled RNA controls, SiRNA against AKT markedly decreased AKT levels).

Figure 6S4: pharmacological inhibitors of EGFR-AKT cascade do not alter the binding of transduced KLF11 to *cPLA2α* promoter: FLO cells were transfected with His-tagged KLF11 and 24 hours later treated with either vehicle or blockers of EGFR-AKT cascade (10 µM PD168393 with 100 µM LY294002). ChIP assays with Omni probe D-8 antibody shows that there was no change in the enrichment of cPLA2α promoter in the blockers treated cells compared to vehicle treated cells suggesting that pharmacological inhibitors of EGFR-AKT cascade do not alter the binding of transduced KLF11 to *cPLA2α* promoter.
